# Predictive Value of C-Reactive Protein (CRP) in Identifying Fatal Outcome and Deep Infections in *Staphylococcus aureus* Bacteremia

**DOI:** 10.1371/journal.pone.0155644

**Published:** 2016-05-16

**Authors:** Tomi Mölkänen, Eeva Ruotsalainen, Esa M. Rintala, Asko Järvinen

**Affiliations:** 1 Division of Infectious Diseases, Inflammation Center, Helsinki University Central Hospital and University of Helsinki, Helsinki, Finland; 2 Department of Hospital Hygiene and Infection Control, Turku University Hospital, Turku, Finland; Hospital de São Francisco Xavier, CHLO, Faculty of Medical Sciences, New University of Lisbon, PORTUGAL

## Abstract

**Introduction:**

Clear cut-off levels could aid clinicians in identifying patients with a risk of fatal outcomes or complications such as deep infection foci in *Staphylococcus aureus* bacteremia (SAB). Cut-off levels for widely used clinical follow-up parameters including serum C-reactive protein (CRP) levels and white blood cell counts (WBC) have not been previously studied.

**Methods:**

430 adult SAB patients in Finland took part in prospective multicentre study in which their CRP levels and WBC counts were measured on the day of the positive blood culture, every other day during the first week, twice a week during hospitalization and at 30 days. Receiver operating characteristic (ROC) analysis was used to evaluate the prognostic value of CRP and WBC on the day of the positive blood culture and at days 4, 7, and 14 in predicting mortality and the presence of deep infections at 30 days. Adjusted hazard ratios (HR) for CRP level and WBC count cut-off values for mortality were calculated by the Cox regression analysis and adjusted odds ratios (OR) for cut-off values to predict the presence of deep infection by the binary logistic regression analysis.

**Results:**

The succumbing patients could be distinguished from the survivors, starting on day 4 after the positive blood culture, by higher CRP levels. Cut-off values of CRP for day 30 mortality in adjusted analysis, that significantly predicted fatal outcome were at day 4 CRP >103 mg/L with sensitivity of 77%, specificity of 55%, and HR of 3.5 (95% CI, 1.2–10.3; p = 0.024), at day 14 CRP >61 mg/L with a sensitivity of 82%, specificity of 80% and HR of 3.6 (95% CI, 1.1–10.3; p<0.039) and cut-off value of WBC at day 14 >8.6 x10^9^/L was prognostic with sensitivity of 77%, specificity of 78% and HR of 8.2 (95% CI, 2.9–23.1; p<0.0001). Cut-off values for deep infection in adjusted analysis were on the day of the positive blood culture CRP >108 mg/L with sensitivity of 77%, specificity of 60%, and HR of 2.6 (95% CI, 1.3–4.9; p = 0.005) and at day 14 CRP >22 mg/L with sensitivity of 59%, specificity of 68%, and HR of 3.9 (95% CI, 1.6–9.5; p = 0.003). The lack of decline of CRP in 14 days or during the second week were neither prognostic nor markers of deep infection focus.

**Conclusions:**

CRP levels have potential for the early identification of SAB patients with a greater risk for death and deep infections.

## Introduction

*Staphylococcus aureus* is one of the most frequent isolates identified in bloodstream infections. Bacteremia due to *S*. *aureus* (SAB) carries high mortality [1,2], and complications such as deep infection foci are found in 60–80% of patients [3–5]. The prognosis in SAB prognosis is mainly determined by the severity of the underlying diseases, immunosuppressive treatment, severity of sepsis and development of deep infections, such as pneumonia [3,6–9]. Prognostic markers could enable the early identification of patients with complications and guide treatment decisions, thereby possibly improving the prognosis.

Numerous biomarkers have been evaluated for potential clinical use, but their use in practice has been scarce [10]. We reported that a new soluble urokinase plasminogen activator receptor (suPAR) could be used to find SAB patients at risk of fatal outcome but could not identify patients with complications such as deep infections [11]. The apoptosis marker cell free-DNA in plasma was recently shown to be associated with a high sequential organ failure assessment (SOFA) score in bacteremic patients [12] and could identify patients with a risk of fatal outcome in intensive care units (ICU) but not in general wards [13]. Procalcitonin (PCT) has been shown to have potential for identifying SAB patients with endocarditis [14,15]. However, no biomarkers that would be helpful in finding other deep infections in SAB patients have been described.

C-reactive protein (CRP) is an acute-phase protein that is widely used in clinical settings. It is rapidly synthesized in hepatocytes following infection, injury or trauma [16]. Increasing CRP concentrations have been shown to be useful for the detection of sepsis or organ dysfunction [16], whereas a rapid decrease in CRP level has been reported to be one of the earliest markers of improvement [17]. CRP has been recognized as a good marker of systemic inflammation and a valuable clinical tool in severe infections [18], but clear cut-off levels to guide clinical decisions in SAB have not been demonstrated. The maximal CRP level might not be useful because it is influenced by genetic variation in the genes responsible for CRP synthesis [19]. This study was undertaken to assess CRP concentrations at various time points during SAB infection that would enable to identify patients with complications. We prospectively followed 430 patients with SAB to evaluate the correlation of CRP levels and for comparison WBC counts on the day of the positive blood culture and at days 4, 7 and 14 thereafter with 30-day mortality and the presence of deep infections.

## Patients and Methods

### Patient population

Four hundred and thirty patients with a blood culture positive for *S*. *aureus* were recruited consecutively from five university hospitals and seven tertiary care hospitals in Finland between 1999 and 2002. [20,21]. The median time between blood culture sampling and study inclusion was 3 days. The trial primarily aimed to examine the potential of two fluoroquinolones (trovafloxacin or levofloxacin) to reduce the high mortality and complications associated with SAB when added to the standard treatment and then patients were randomly assigned to receive standard antibiotic therapy or that together with a fluoroquinolone. Thereafter, all patients were prospectively followed for 3 months and the study treatments were open. Parenteral cloxacillin or dicloxacillin were used as the standard antibiotic therapy in 327 out of 430 (76%) patients. All other antimicrobials were provided less frequently administered [cefuroxime in 80 (19%) patients and vancomycin in 8 (2%) patients]. Rifampicin was added only in cases of deep infection and was given to 306 (71%) patients.

The exclusion criteria were bacteremia due to methicillin-resistant *S*. *aureus* (MRSA) (n = 5), age younger than 18 years, proven or suspected pregnancy, breastfeeding, epilepsy, another bacteremia during the previous 28 days, polymicrobial bacteremia (≥ 3 microbes), history of allergy to any quinolone antibiotic, previous tendinitis during fluoroquinolone therapy, prior fluoroquinolone use for more than 5 days before randomization, positive culture for *S*. *aureus* only from a central intravenous catheter, neutropenia (<0.5 x 10^9^ L^-1^), failure to supply informed consent, meningitis or imprisonment. Each patient was recruited into the study only once to avoid double inclusion in cases with repeated bacteremia. The protocol was approved by the ethics committees of all study sites. Written informed consent was obtained from all patients or their representatives. All patients were treated with an antimicrobial agent effective against the *S*. *aureus* strain isolated in vitro beginning on the day of the positive blood culture.

### Definitions

Intravenous drug users (IDUs) were defined as patients who had injected drugs within the past 6 months prior to randomization based on a history taken upon admission. SAB was hospital-acquired if the first positive blood culture was obtained ≥48 hours after admission or the patient was a resident in a long-term care facility or had attended hemodialysis within the preceding 2 months. The prognosis and severity of underlying diseases were characterized as healthy, nonfatal, ultimately or rapidly fatal disease according to the criteria of McCabe and Jackson [22]. The infection foci were documented based on clinical, bacteriological, radiological, or pathological investigations. Endocarditis was defined by modified Duke criteria [23]. Deep infection foci included, endocarditis, pneumonia, deep-seated abscess, osteomyelitis, septic arthritis, mediastinitis, and any foreign-body infection. Definitive deep infection foci were verified by bacteriological, radiological or pathological investigations but they were regarded as suspected if it was evident from clinical findings only. Definition of severe sepsis was sepsis in combination with hypotension, hypoperfusion or organ failure [24]. Mortality was recorded at 30 days.

### Analytical methods

Blood samples for CRP measurement and white blood cell counts (WBC counts) were routinely collected on the day of the positive blood culture for *S*. *aureus* from all patients. Thereafter, samples were collected every other day during the first week, twice a week during hospitalization and at 28 days. Serum concentrations of CRP and WBC counts were measured in the study site laboratory using standard laboratory methods. The serum or plasma was subjected to automatic immunoturbidimetric analysis using the 917 or Modular PP-analyzer (Hitachi Ltd, Tokyo, Japan) and Tina-quant CRP reagents (Roche Diagnostics, Tina-quant CRP). The normal value of the CRP concentration was <10 mg/L for both methods.

### Statistical analysis

First, a test for normality was performed and parametric tests were chosen when possible. The associations between categorical variables were analyzed by Pearson’s χ2-test or Fisher’s exact test as appropriate. Odds ratios (ORs) with 95% confidence intervals (CIs) were determined to estimate the significance of differences between the groups. All tests were two-tailed and p<0.05 was considered significant. Hazard ratios (HR) of CRP and WBC count cut-off values for 30-day mortality were calculated by the Cox regression analysis adjusted with the prognostic factors. Adjusted Odds ratios (OR) for factors associated with deep infection foci were analyzed by binary logistic regression analysis. In both multivariable analyses, forward selection was performed using the Akaike information criteria (AIC) [25] which includes significant covariates with p<0.20. Receiver operating characteristic (ROC) analysis was used to evaluate the prognostic value of CRP and WBC in predicting 30-day mortality and the presence of deep infections. Abnormal leukocytes were analyzed by the χ2-test. The area under the curve (AUC) was calculated for each ROC. The cut-off points for general optimal tests were chosen to optimize the rate of true positives whilst minimizing the rate of false positives. SPSS^®^ version 20.0 (SPSS Inc., Chicago, IL, USA) was used for data analysis.

## Results

### Clinical outcome and deep infections

Fifty-three cases (12%) out of 430 patients with SAB had a fatal outcome within 30 days after the positive blood culture. Table 1 shows the results of both univariate and multivariate analysis for characteristics, predisposing factors and severity of illness stratified according to 30-day mortality. Age >60 years, chronic alcoholism, fatal underlying disease, chronic renal failure, severe sepsis during the first 3 days, pneumonia or endocarditis were found as significant prognostic factors in multivariate analysis. Deep infection focus was diagnosed already at day 3 after the positive blood culture in 325 (75%) patients. Within 30-day follow-up a deep focus was found in total in 351 patients (82%), including a deep seated abscess in 185 (43%) patients, osteomyelitis in 141 (33%) patients, pneumonia in 152 (35%) patients, infection of a foreign body in 79 (18%) patients, endocarditis in 74 (17%) patients, and septic arthritis in 56 (13%) patients.

**Table 1:** Characteristics, predisposing factors and severity of illness in 430 patients with *Staphylococcus aureus* bacteremia (SAB) stratified according to 30-day mortality. OR = odds ratio for fatal outcome. HR = hazard ratio for fatal outcome, 95% CI = 95% confidence interval. All values are given as number of patients (%).

	Univariate analysis				Multivariate analysis^f^	
	Fatalitiesn = 53 (12)	Survivors n = 377 (88)	OR (95% CI)	p- value	HR (95% CI)	p-value
Age >60 years	43 (80)	174 (46)	5.0 (2.4–10.3)	<0.0001	4.2 (2.0–8.6)	<0.0001
Male sex	33 (62)	235 (62)	1.0 (0.6–1.8)	1.000	-	-
Healthcare-associated SAB	32 (60)	200 (53)	1.3 (0.8–2.4)	0.378	-	-
Foreign body^b^	12 (23)	100 (27)	0.8 (0.4–1.6)	0.619	-	-
Intravenous drug abuse^a^	1 (2)	43 (11)	0.15 (0.0–1.1)	0.029	-	-
Immunosuppressive treatment	16 (30)	42 (11)	3.5 (1.8–6.7)	<0.0001	1.8 (0.9–3.3)	0.087
Chronic alcoholism	11 (21)	37 (10)	2.4 (1.1–5.1)	0.032	2.7 (1.2–6.1)	0.017
Diabetes	13 (25)	95 (25)	1.0 (0.5–1.9)	1.000	0.5 (0.3–1.19	0.090
Chronic renal failure^c^	14 (26)	46 (12)	2.6 (1.3–5.1)	0.010	2.3 (1.1–5.1)	0.037
Liver disease	7 (13)	60(16)	0.8 (0.3–1.9)	0.691	-	-
Malignancy	13 (25)	50 (13)	2.1 (1.0–4.3)	0.038	-	-
HIV	1 (2)	7 (2)	1.0 (0.1–8.2)	1.000	-	-
Ultimately or rapidly fatal disease^d^	31 (59)	88 (23)	4.6 (2.5–8.4)	<0.0001	2.1 (1.1–4.0)	0.033
Severe sepsis^e^	12 (23)	25 (7)	4.1 (1.9–8.8)	0.001	2.1 (1.0–4.3)	0.054
Any deep infection	51 (96)	300 (80)	6.5 (1.6–27.5)	0.002	4.1 (0.9–18.4)	0.065
Deep seated abscess	23 (43)	162 (43)	1.0 (0.6–1.8)	1.000	-	-
Osteomyelitis	17 (32)	124 (33)	1.0 (0.5–1.6)	1.000	-	-
Pneumonia	34 (64)	118 (31)	3.9 (2.2–7.2)	<0.0001	2.0 (1.1–3.7)	0.033
Foreign body infection	9 (17)	70 (19)	0.9 (0.4–1.9)	1.000	-	-
Endocarditis	17 (32)	57 (15)	2.6 (1.4–4.9)	0.006	2.5 (1.4–4.7)	0.004
Septic arthritis	4 (8)	52 (14)	0.5 (0.2–1.5)	0.276	-	-

^a^Injection drug use within 6 months.

^b^Foreign body implanted within a year.

^c^Constantly elevated serum creatinine (≥180 μmol/l).

^d^According to the criteria of McCabe and Jackson [22].

^e^Severe sepsis during the 3 days from the blood culture time point.

^f^ Cox regression analysis -2 log likelihood was 547.718 and model chi-square 100.102, p<0.0001

### Predictors of a fatal outcome

**Fig 1:**
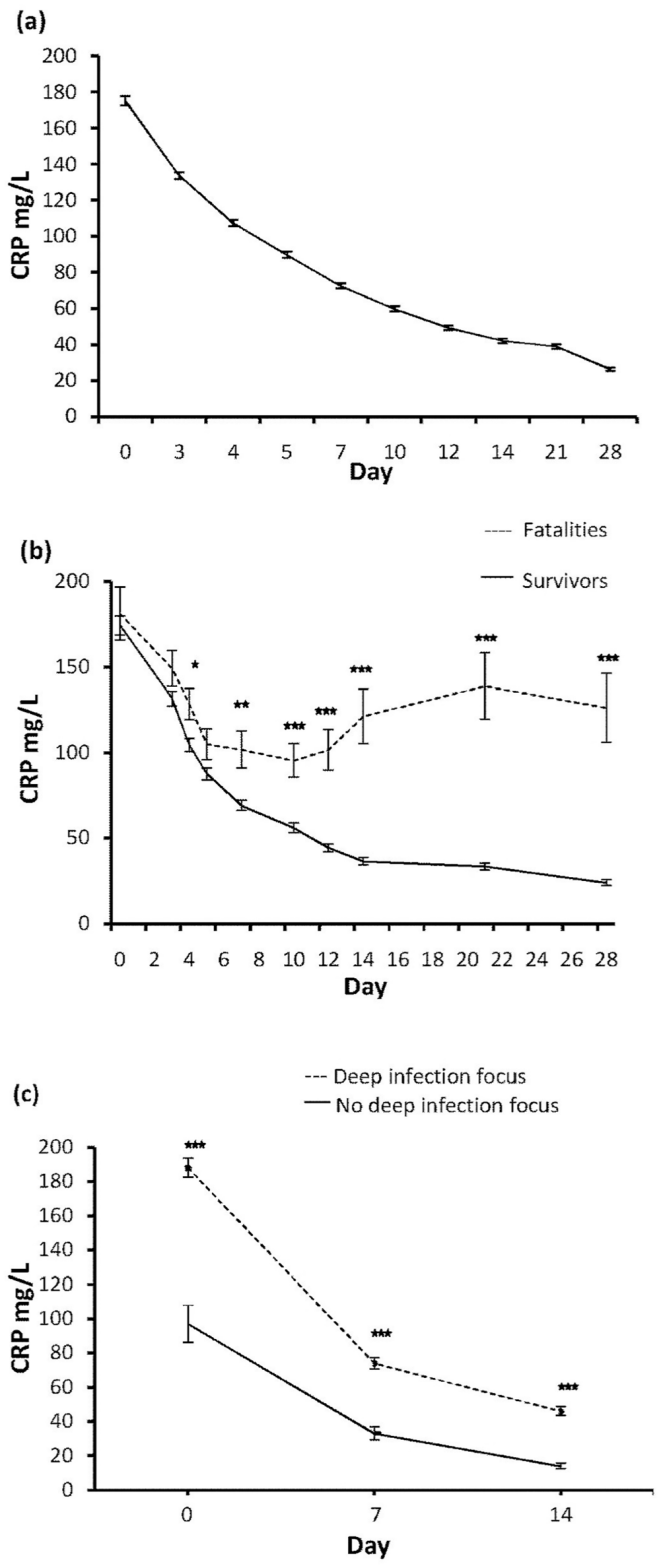
C-reactive protein (CRP) (Mean ±SEM) levels in patients with *Staphylococcus aureus* bacteremia (SAB) within 30 days of the positive blood culture. (A) All 430 SAB patients. (B) SAB patients with a fatal outcome within 30 days (n = 53) and survivors (n = 377). (C) Mean CRP levels stratified according to the presence (n = 351) or absence (n = 79) of deep infection focus. Stars indicate p-values of the Student’s T-test. * p<0.05, ** p<0.01 and *** p<0.001.

The CRP concentration was in an average highest on the day of the positive blood culture and declined gradually thereafter as shown in Fig 1A. In cases with fatal outcome, the mean CRP level differed from the survivors by day 4, and the difference remained significant throughout the observation period as shown in Fig 1B. The median CRP level in fatal cases at day 4 was 127 mg/L (interquartile range [IQR], defined as the difference between the third and the first quartile, 97 mg/L), which was higher than the median value in the survivors (91 mg/L, IQR, 96 mg/L, p = 0.005).

**Fig 2:**
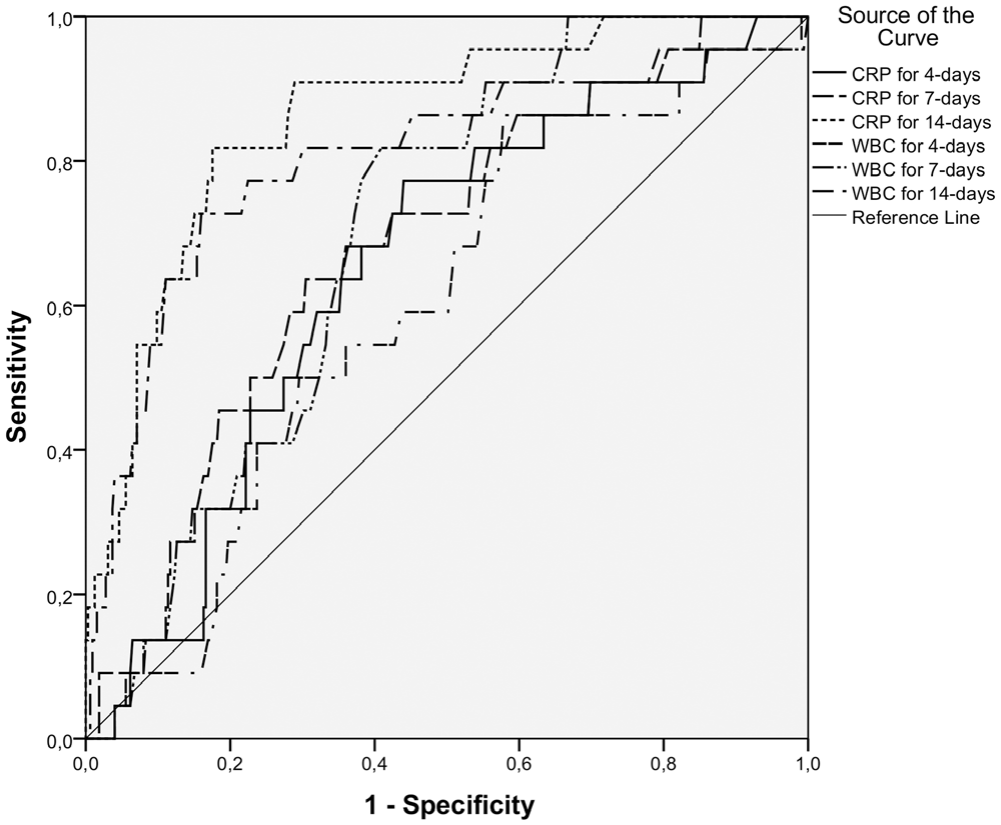
Receiver-operating characteristic (ROC) curve analyses of C-reactive protein (CRP) concentrations and white blood cell counts (WBC) with respect to 30-day mortality in *Staphylococcus aureus* bacteremia (n = 430). The area under the curve (AUC) for CRP on the day 4 was 0.65 (95% CI, 0.55–0.76; p = 0.016) with a cut-off value of 104 mg/L with sensitivity of 77% and specificity of 55%. For CRP on the day 7 the AUC was 0.68 (95% CI, 0.58–0.79; p = 0.004) with a cut-off value of 66 mg/L with sensitivity of 73% and specificity of 55% and for CRP on day 14 the AUC was 0.86 (95% CI, 0.79–0.94; p<0.0001) with a cut-off value of 61mg/L with sensitivity of 82% and specificity of 80%. The corresponding AUC for WBC on the day 4 was 0.60 (95% CI, 0.49–0.71; p = 0.116) with a cut-off value of 8.0 x10^9^/L with sensitivity of 86% and specificity of 41%. For WBC on the day 7 the AUC was 0.70 (95% CI, 0.62–0.78; p = 0.002) with a cut-off value of 9.8 x10^9^/L with sensitivity of 77% and specificity of 62%. For WBC on the day 14 the AUC was 0.80 (95% CI, 0.70–0.92; p<0.0001) with a cut-off value of 8.6 x10^9^/L with sensitivity of 77% and specificity of 78%.

The CRP levels at day 4, 7, and 14 after the positive blood culture were found to be significantly associated with 30-day mortality based on the ROC analysis whereas WBC counts were significant in regard to mortality only at days 7 and 14 as shown in Fig 2. Optimal cut-off value of CRP at day 4 was: 104 mg/L with sensitivity of 77% and specificity of 55%, at day 7day CRP 66 mg/L with sensitivity of 73% and specificity of 55% and at 14 day CRP 61mg/L with sensitivity of 82% and specificity of 80%. For WBC count optimal cut-off value for was 9.8 x10^9^/L at day 7 with sensitivity of 77% and specificity of 62% and 8.6 x10^9^/L at day 14 with sensitivity of 77% and specificity of 78%. At the time of the positive blood culture, the CRP levels and WBC counts could not significantly predict patients with fatal outcome.

Univariate analysis calculated for CRP and WBC optimal cut-off values gave odds ratios for 30-day mortality as shown in Table 2. All CRP cut-off levels were found as significant predictors for mortality in univariate analysis. In univariate analysis. WBC count cut-off at day 7 of 9.8 x10^9^/L had an OR of 3.6 (95% CI, 1.8–7.0; p<0.0001) when also low WBC count levels were taken into account by using a combined parameter with a WBC <4.5 or >9.8 x10^9^/L. Prognostic factors, comprising characteristics, predisposing factors and severity of illness, from Table 1, were analysed in multivariate analysis together with the cut-off values of CRP and WBC at days 4, 7 and 14 and the results are shown in Table 2. The cut-off values for CRP at day 4 >103 mg/L, at day 14 > 61 mg/L and fall of CRP concentration less than by 50% in 14 days were all markers of poor prognosis also in multivariate analysis. Overall -2 log likelihood of the predictive power of the multivariable model including the CRP and WBC count cut-off values (Table 2) was (-2 log likelihood 547.718, chi-square 100.102, p<0.0001) and it was superior as compared to the model (Table 1) without them (-2 log likelihood 547.718, chi-square 100.102, p<0.0001)

**Table 2:** Cut-off values for C-reactive protein (CRP) levels (mg/L) and white blood cell counts (WBC) in finding patients with fatal outcome among 430 patients with *Staphylococcus aureus* bacteremia. OR = odds ratio for fatal outcome. HR = hazard ratio for fatal outcome, 95% CI = 95% confidence interval. All values are given as number of patients (%).

	Univariate analysis				Multivariate analysis^a^	
Cut-off value	Fatalities n = 53 (12)	Survivors n = 377 (88)	OR 95% (CI)	p-value	HR (95%CI)	p-value
CRP >103 mg/L at day 4	33 (66)	158 (43)	2.6 (1.4–4.8)	0.004	3.5 (1.2–10.3)	0.024
CRP >66 mg/L at day 7	27 (66)	155 (43)	2.6 (1.3–5.1)	0.005	-	-
CRP >61 mg/L at day 14	21 (84)	74 (21)	19.8 (6.6–59.4)	<0.0001	3.6 (1.1–10.3)	0.039
Fall of CRP <50% in 14 days	13 (52)	56 (16)	5.7 (2.5–13.1)	<0.0001	2.0 (0.8–5.4)	0.148
Fall of CRP <50% between 7 to 14 days	20 (87)	151 (44)	8.5 (2.5–29.2)	<0.0001	-	-
WBC <4.5 or >10.3 x109/L at day 4	32 (63)	166 (45)	2.0 (1.1–3.7)	0.024	-	-
WBC <4.5 or >9.8 x10^9^/L at day 7	34 (81)	153 (42)	5.8 (2.6–12.8)	<0.0001	-	-
WBC >8.6 x10^9^/L at day 14	17 (65)	80 (23)	6.5 (2.8–15.0)	<0.0001	8.2 (2.9–23.1)	<0.0001
Age >60 years	43 (80)	174 (46)	5.0 (2.4–10.3)	<0.0001	3.6 (1.1–11.5)	0.029
Malignancy	13 (25)	50 (13)	2.1 (1.0–4.3)	0.038	0.3 (0.1–1.2)	0.087
Ultimately or rapidly fatal disease^b^	31 (59)	88 (23)	4.6 (2.5–8.4)	<0.0001	6.5 (2.4–17.6)	<0.0001
Pneumonia	34 (64)	118 (31)	3.9 (2.2–7.2)	<0.0001	8.1 (2.5–25.8)	<0.0001

^a^Cox regression analysis -2 log likelihood was 169.320 and chi-square 92.437, p<0.0001. All factors from Table 1 were included in the analysis.

^b^According to the criteria of McCabe and Jackson [22].

### Predictors of deep infection foci

The CRP levels in patients with a deep infection focus compared to those without a deep infection was significantly higher on the day of the positive blood culture and throughout the 30-day period as shown in Fig 1C.

**Fig 3:**
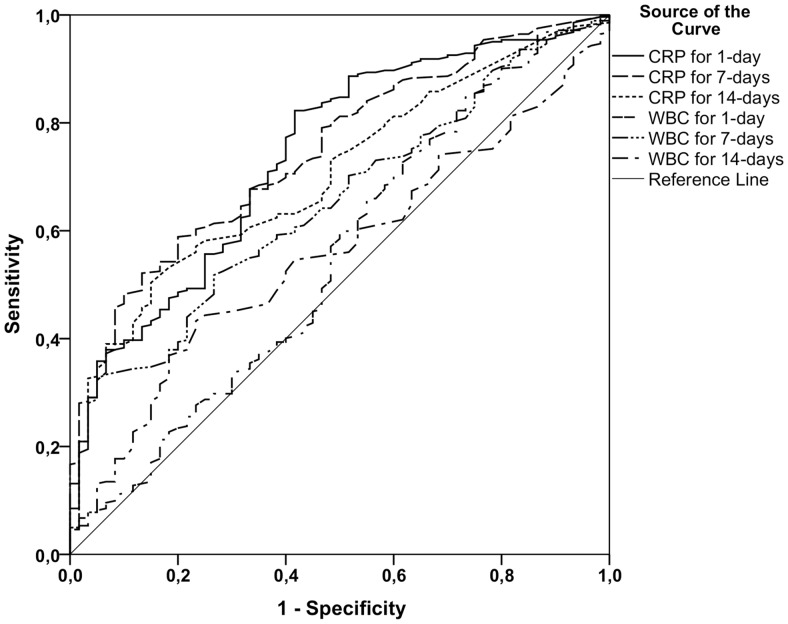
Receiver-operating characteristic (ROC) curve analyses of C-reactive protein (CRP) concentrations and white blood cell counts (WBC) with respect to presence of any deep infection focus recorded during 30-days. The area under the curve (AUC) for the day of the positive blood culture, CRP was 0.074 (95% CI, 0.67–0.81; p<0.0001) with a cut-off value of 108 mg/L with sensitivity of 77% and specificity of 60%. For CRP on day 7, the AUC was 0.75 (95% CI, 0.68–0.81; p<0.0001) with a cut-off value of 44 mg/L with sensitivity of 68% and specificity of 67% and for CRP on day 14 the AUC was 0.70 (95% CI, 0.64–0.77; p<0.0001) with a cut-off value of 22 mg/L with sensitivity of 59% and specificity of 68%. The corresponding AUC for WBC on the day 1 was 0.55 (95% CI, 0.46–0.63; p = 0.236) and a cut-off value was not determined. For WBC on day 7 the AUC was 0.65 (95% CI, 0.58–0.72; p<0.0001) with a cut-off value of 8.5 x109/L with sensitivity of 59% and specificity of 62%. For WBC on 14 days, the AUC was 0.56 (95% CI, 0.49–0.63; p = 0.153) with a cut-off value of 7.25 x109/L with sensitivity of 44% and specificity of 75%.

The CRP levels on the day of the positive blood culture and at days 7 and 14 after that were significantly associated with the presence of any deep infection during the 30-day surveillance period whereas WBC counts were significant only at day 7. ROC curve analysis results are shown in Fig 3. The optimal CRP cut-off value for screening a deep infection focus was 108 mg/L at day 1 with sensitivity of 77% and specificity of 60%, 44 mg/L at day 7 with sensitivity of 68% and specificity of 67%, and 22 mg/L at day 14 with sensitivity of 59% and specificity of 76%. The optimal WBC count cut-off value at day 7 was 8.5 x10^9^/L with sensitivity of 59% and specificity of 62%. On the day of the positive blood culture and at day 14 WBC count were not significant.

Table 3 shows all clinical factors associated by univariate analysis with the presence of deep infection. Multivariate analysis of all these clinical factors and CRP and WBC cut-off values indicates that an intravenous drug abuse was the strongest predictor for the presence of any deep infection focus with OR 11.9 (95% CI, 1.5–93.2; p = 0.018) and CRP >108 mg/L at day 1 with OR 2.6 (95% CI, 1.3–4.9; p = 0.005) and CRP >22 mg/L at day 14 with OR 3.9 (95% CI, 1.6–9.5; p = 0.003) were the second strongest. CRP at day 7 >44 mg/L and change of CRP<50% within 14 days or during the second week and all WBC count cut-off values were non-significant.

**Table 3:** Factors associated with a deep infection focus in 430 patients with *Staphylococcus aureus* bacteremia. OR = odds ratio for presence of deep infection, 95% CI = 95% confidence interval. All values are given as number of patients (%).

	Univariate analysis				Multivariate analysis^a^	
	Deep infection n = 351 (82)	No deep infection n = 79 (18)	OR (95% CI)	p-value	OR (95%CI)	p-value
Male sex	224 (84)	44 (56)	1.4 (0.86–2.3)	0.199	-	-
Hospital-acquired	178 (51)	54 (68)	0.48 (0.3–0.80)	0.006	-	-
Foreign body^b^	82 (23)	30 (38)	0.50 (0.3–0.8)	0.010	-	-
IDU^c^	43 (12)	1 (1)	11 (1.5–80.3)	0.002	11.9 (1.5–93.2)	0.018
Chronic renal failure^d^	42 (12)	18 (23)	0.5 (0.2–0.9)	0.064	0.47 (0.2–1.0)	0.064
Malignancy	49 (78)	14 (22)	0.8 (0.4–1.4)	0.383	-	-
CRP >108 mg/L at day 1	264 (76)	31 (39)	4.9 (2.9–8.2)	<0.0001	2.6 (1.3–4.9)	0.005
CRP >72 mg/L at day 4	229 (68)	30 (39)	3.4 (2.0–5.6)	<0.0001	-	-
CRP >44 mg/L at day 7	223 (67)	20 (27)	5.7 (3.2–10)	<0.0001	2.0 (1.0–4.3)	0.063
CRP >22 mg/L at day 14	177 (57)	16 (23)	4.4 (2.4–8.1)	<0.0001	3.9 (1.6–9.5)	0.003
Fall of CRP <50% in 14 days	53 (17)	16 (23)	0.7 (0.4–1.3)	0.301	-	-
Fall of CRP <50% during the second week	136 (46)	35 (52)	0.8 (0.5–1.3)	0.420	0.5 (0.2–1.0)	0.064
WBC >8.0 x10^9^/L at day 4	215 (63)	34 (44)	2.2 (1.4–3.7)	0.002	-	-
WBC >8.5 x10^9^/L at day 7	189 (57)	27 (37)	2.4 (1.4–4.0)	0.001	-	-
WBC >7.25 x10^9^/L at day 14	139 (45)	19 (28)	2.1 (1.2–3.8)	0.010	-	-

Deep infection occurred at any time within the 30-day follow-up.

^a^Binary logistic regression analysis results

^b^Foreign body implanted within a year

^c^Intravenous drug abuse during the 6 months preceding the positive blood culture.

^d^Chronically elevated serum creatinine (≥180 μmol/l).

## Discussion

CRP and WBC counts are usually followed during the treatment of serious infections such as SAB. Constantly elevated values are usually regarded as alarming signals, but clear cut-off points to aid in clinical decisions have not been identified. Validated tests with clearly defined limits might save costs if they could help to guide treatment efforts and examinations to the right patients and simplify the treatment of patients with a low risk for fatal outcomes or complications.

We observed that the decline of CRP deviated in patients with fatal outcome compared to the survivors already on the 4^th^ day after the positive blood culture. In addition, abnormal WBC counts and CRP at the end of the second week significantly predicted a risk of fatal outcome within 30 days. The CRP levels and WBC counts were both significant predictors of fatal outcome when adjusted for the identified risk factors for fatal outcome in the multivariate analysis. Furthermore, the CRP level was higher among patients with a deep infection focus already on the day of the positive blood culture and at 2 weeks compared to patients in whom a deep infection focus was not verified.

Both CRP and WBC seemed to predict the risk for fatal outcome fairly well. The ROC-curve analysis suggested that CRP might be a slightly better prognostic marker due to its higher AUC value, but the cut-off values for CRP (over 66 mg/L) and WBC (over 9.8 x 10^9^/L) at one week had fairly similar sensitivities (0.73–0.77) and specificities (0.55–0.62), suggesting approximately equal clinical accuracy. However, when all other risk factors for fatal outcome were taken into account only CRP at day 4 (over 103 mg/L) and at the end of the second week both CRP (over 61 mg/L) and WBC (over 8.6x10^9^/L) remained significant.

In accordance with our results, CRP levels were observed to correlate with fatal outcome in sepsis [26] and in severe community acquired pneumonia [27–29]. However, contrasting results have also been published for bacteremia patients with no association of CRP levels with fatal outcome [30]. CRP was not observed to predict hospital mortality in sepsis patients [31], and CRP levels on the day of sepsis diagnosis poorly predicted survival [32]. In contrast to our findings, these studies included various causes of systemic inflammatory response (SIRS) and included both patients with an infection and non-infectious causes whereas we included only bacteremic patients with sepsis and further focused on only one causative agent. This difference in results suggests that the particular cut-off points for fatal outcome might be determined specifically for each disease.

Mortality in SAB is high but variable in recent studies [9,33–35]. In our study, the mortality at 30 days was 12%, which was at the lower end of the mortality range reported in recent publications [33,35]. All our patients received effective antimicrobial therapy from the day of the positive blood culture, and we did not include any cases due to MRSA. We may have missed some of the most serious cases due to problems in enrolling them into the study but patients treated both in the general ward and in intensive care unit were recruited. The treatment of septic patients has certainly changed since this patient material was collected but the mortality in SAB is virtually unchanged. The low mortality in our patient material suggests that the results might be extrapolated even into current treatment practice. The low mortality in our study may have decreased the power to detect the predictive value of CRP levels and WBC counts for fatal outcome and may also have affected the extrapolation of cut-off values to other patient groups.

The prognosis of SAB is significantly affected by the severity of the underlying diseases [8] and the presence of deep infection foci or complications such as endocarditis, osteomyelitis or foreign body infections [3,36]. Intravenous antibiotic treatment is recommended for 4–6 weeks in SAB patients with a deep infection focus, and the relatively high recurrence of SAB (9–23%) could be partially explained by unidentified deep infections [4,37–40]. A meticulous search for deep infections may improve the prognosis and is best achieved by an infectious disease specialist consultation [9,35,41,42]. Therefore, inexpensive markers to detect the presence of deep infections might be useful to guide diagnostic procedures or limit support that might be not needed.

CRP seemed to be of greater value than WBC in predicting the presence of a deep infection focus. CRP levels higher than 108 mg/L on the day of the positive blood culture significantly suggested the presence of a deep infection focus with an acceptable sensitivity of 0.77. CRP levels greater than 44 mg/L at one week and 22 mg/L at two weeks were also significant prognostic markers for a deep infection focus with a somewhat higher specificity compared to the CRP level on the day of the positive blood culture. Moreover, an elevated WBC count detected patients with a deep infection focus in univariate analysis but these differences were not significant in the multivariate analysis.

Few other biomarkers have been evaluated for the detection of deep infection focus. In our earlier publication, the soluble urokinase receptor (suPAR) was prognostic for fatal outcome in SAB patients but it was not helpful in finding deep infections [11]. The erythrocyte sedimentation rate (ESR) was sensitive but nonspecific for the detection of osteomyelitis [43,44]. Procalcitonin (PCT) was shown to be sensitive and useful for the differential diagnosis of infectious or non-infectious inflammatory processes in various conditions [45]. However, the use of PCT to detect deep infections in SAB patients has not been studied. Cell-free DNA in plasma was observed to be a good prognostic marker of fatal outcome among SAB patients treated in intensive care units but did not predict the presence of a deep infection focus [13].

We previously reported that the maximal CRP in SAB was affected by genetic polymorphisms in the genes regulating CRP synthesis [19]. In accordance, the CRP on the day of the positive blood culture was not a significant prognostic marker for fatal outcome. Because the CRP level might be individually set, we also analyzed the prognostic value of the relative decline in the CRP level during the first two weeks. The lack of a 50% decrease in the CRP level in the first two weeks (OR 8.2) or during the second week (OR 9.5) were both predictors of a fatal outcome in univariate analysis, but not in multivariate analysis. This finding suggests that clinicians should not only look for the absolute cut-off values but also follow the decline of CRP when assessing the risk for a fatal outcome in individual patients. Interestingly, the lack of decline of CRP was not observed to be a predictor of a deep infection focus. This finding suggests that the presence of a deep infection could lead to a higher level of inflammation as measured by higher CRP levels, but the CRP level would also decline concomitantly in cases with a deep infection focus.

In conclusion, we evaluated the prognostic impact of two widely followed inexpensive laboratory markers for SAB, which is one of the most common bacteremias. CRP levels higher than 103 mg/L at day 4 and higher than 61 mg/L at day 14 were significant markers for a fatal outcome like WBC over 8.6 x 10^9^/L or lower than 4.5 x 10^9^/ at day 14. The presence of a deep infection was predicted by a CRP level greater than 108 mg/L already on the day of the positive blood culture and by CRP greater than 22 mg/L at two weeks. These cut-off values might be helpful in guiding clinicians treating patients with SAB

## Supporting Information

S1 Dataset Minimal data set. (SAV)

## References

[1] Shorr AF, Tabak YP, Killian AD, Gupta V, Liu LZ, Kollef MH. Healthcare-associated bloodstream infection: A distinct entity? Insights from a large U.S. database. Crit Care Med. 2006;34: 2588–2595. 10.1097/01.CCM.0000239121.09533.09 16915117

[2] Wolkewitz M, Frank U, Philips G, Schumacher M, Davey P, BURDEN Study Group. Mortality associated with in-hospital bacteraemia caused by Staphylococcus aureus: a multistate analysis with follow-up beyond hospital discharge. J Antimicrob Chemother. 2011;66: 381–386. 10.1093/jac/dkq424 21098543

[3] Mylotte JM, Tayara A. Staphylococcus aureus bacteremia: predictors of 30-day mortality in a large cohort. Clin Infect Dis. 2000;31: 1170–1174. 11073748 10.1086/317421

[4] Fowler VG Jr, Olsen MK, Corey GR, Woods CW, Cabell CH, Reller LB, et al Clinical identifiers of complicated Staphylococcus aureus bacteremia. Arch Intern Med. 2003;163: 2066–2072. 14504120 10.1001/archinte.163.17.2066

[5] Fatkenheuer G, Preuss M, Salzberger B, Schmeisser N, Cornely OA, Wisplinghoff H, et al Long-term outcome and quality of care of patients with Staphylococcus aureus bacteremia. Eur J Clin Microbiol Infect Dis. 2004;23: 157–162. 14986158 10.1007/s10096-003-1083-3

[6] Benfield T, Espersen F, Frimodt-Moller N, Jensen AG, Larsen AR, Pallesen LV, et al Increasing incidence but decreasing in-hospital mortality of adult Staphylococcus aureus bacteraemia between 1981 and 2000. Clin Microbiol Infect. 2007;13: 257–263. 17391379 10.1111/j.1469-0691.2006.01589.x

[7] Jacobsson G, Gustafsson E, Andersson R. Outcome for invasive Staphylococcus aureus infections. Eur J Clin Microbiol Infect Dis. 2008;27: 839–848. 10.1007/s10096-008-0515-5 18449584

[8] Forsblom E, Ruotsalainen E, Molkanen T, Ollgren J, Lyytikainen O, Jarvinen A. Predisposing factors, disease progression and outcome in 430 prospectively followed patients of healthcare- and community-associated Staphylococcus aureus bacteraemia. J Hosp Infect. 2011 10.1016/j.jhin.2011.03.010 21511366

[9] Rieg S, Peyerl-Hoffmann G, de With K, Theilacker C, Wagner D, Hubner J, et al Mortality of S. aureus bacteremia and infectious diseases specialist consultation—a study of 521 patients in Germany. J Infect. 2009;59: 232–239. 10.1016/j.jinf.2009.07.015 19654021

[10] Pierrakos C, Vincent JL. Sepsis biomarkers: a review. Crit Care. 2010;14: R15 10.1186/cc8872 20144219 PMC2875530

[11] Molkanen T, Ruotsalainen E, Thorball CW, Jarvinen A. Elevated soluble urokinase plasminogen activator receptor (suPAR) predicts mortality in Staphylococcus aureus bacteremia. Eur J Clin Microbiol Infect Dis. 2011 10.1007/s10096-011-1236-8 21479972

[12] Huttunen R, Syrjanen J, Vuento R, Laine J, Hurme M, Aittoniemi J. Apoptosis markers soluble Fas (sFas), Fas Ligand (FasL) and sFas/FasL ratio in patients with bacteremia: a prospective cohort study. J Infect. 2012;64: 276–281. 10.1016/j.jinf.2011.12.006 22207003

[13] Forsblom E, Aittoniemi J, Ruotsalainen E, Helmijoki V, Huttunen R, Jylhava J, et al High cell-free DNA predicts fatal outcome among Staphylococcus aureus bacteraemia patients with intensive care unit treatment. PLoS One. 2014;9: e87741 10.1371/journal.pone.0087741 24520336 PMC3919733

[14] Cuculi F, Toggweiler S, Auer M, der Maur C, Zuber M, Erne P. Serum procalcitonin has the potential to identify Staphylococcus aureus endocarditis. Eur J Clin Microbiol Infect Dis. 2008;27: 1145–1149. 10.1007/s10096-008-0541-3 18521635

[15] Knudsen JB, Fuursted K, Petersen E, Wierup P, Molgaard H, Poulsen SH, et al Procalcitonin in 759 patients clinically suspected of infective endocarditis. Am J Med. 2010;123: 1121–1127. 10.1016/j.amjmed.2010.07.018 20870199

[16] Black S, Kushner I, Samols D. C-reactive Protein. J Biol Chem. 2004;279: 48487–48490. 15337754 10.1074/jbc.R400025200

[17] Povoa P, Coelho L, Almeida E, Fernandes A, Mealha R, Moreira P, et al Early identification of intensive care unit-acquired infections with daily monitoring of C-reactive protein: a prospective observational study. Crit Care. 2006;10: R63 16635270 10.1186/cc4892 PMC1550913

[18] Lobo SM, Lobo FR, Bota DP, Lopes-Ferreira F, Soliman HM, Melot C, et al C-reactive protein levels correlate with mortality and organ failure in critically ill patients. Chest. 2003;123: 2043–2049. 12796187 10.1378/chest.123.6.2043

[19] Molkanen T, Rostila A, Ruotsalainen E, Alanne M, Perola M, Jarvinen A. Genetic polymorphism of the C-reactive protein (CRP) gene and a deep infection focus determine maximal serum CRP level in Staphylococcus aureus bacteremia. Eur J Clin Microbiol Infect Dis. 2010 10.1007/s10096-010-0978-z 20552244

[20] Ruotsalainen E, Jarvinen A, Koivula I, Kauma H, Rintala E, Lumio J, et al Levofloxacin does not decrease mortality in Staphylococcus aureus bacteraemia when added to the standard treatment: a prospective and randomized clinical trial of 381 patients. J Intern Med. 2006;259: 179–190. 16420547 10.1111/j.1365-2796.2005.01598.x

[21] Ruotsalainen E, Sammalkorpi K, Laine J, Huotari K, Sarna S, Valtonen V, et al Clinical manifestations and outcome in Staphylococcus aureus endocarditis among injection drug users and nonaddicts: a prospective study of 74 patients. BMC Infect Dis. 2006;6: 137 16965625 10.1186/1471-2334-6-137 PMC1584240

[22] McCabe WR J G. Gram-negative bacteremia. I. Etiology and ecology. 1962;Arch Intern Med 110: 847–855.

[23] Li JS, Sexton DJ, Mick N, Nettles R, Fowler VG Jr, Ryan T, et al Proposed modifications to the Duke criteria for the diagnosis of infective endocarditis. Clin Infect Dis. 2000;30: 633–638. 10770721 10.1086/313753

[24] Levy MM, Fink MP, Marshall JC, Abraham E, Angus D, Cook D, et al 2001 SCCM/ESICM/ACCP/ATS/SIS International Sepsis Definitions Conference. Crit Care Med. 2003;31: 1250–1256. 12682500 10.1097/01.CCM.0000050454.01978.3B

[25] Akaike H. A New Look at the Statistical Model Identification. 1974;19(6): 716–723.

[26] Yamamoto S, Yamazaki S, Shimizu T, Takeshima T, Fukuma S, Yamamoto Y, et al Prognostic utility of serum CRP levels in combination with CURB-65 in patients with clinically suspected sepsis: a decision curve analysis. BMJ Open. 2015;5: e007049-2014-007049. 10.1136/bmjopen-2014-007049 PMC4420934 25922102

[27] Oberhoffer M, Karzai W, Meier-Hellmann A, Bogel D, Fassbinder J, Reinhart K. Sensitivity and specificity of various markers of inflammation for the prediction of tumor necrosis factor-alpha and interleukin-6 in patients with sepsis. Crit Care Med. 1999;27: 1814–1818. 10507603 10.1097/00003246-199909000-00018

[28] Chalmers JD, Singanayagam A, Hill AT. C-reactive protein is an independent predictor of severity in community-acquired pneumonia. Am J Med. 2008;121: 219–225. 10.1016/j.amjmed.2007.10.033 18328306

[29] Povoa P, Teixeira-Pinto AM, Carneiro AH, Portuguese Community-Acquired Sepsis Study Group SACiUCI. C-reactive protein, an early marker of community-acquired sepsis resolution: a multi-center prospective observational study. Crit Care. 2011;15: R169 10.1186/cc10313 21762483 PMC3387609

[30] Huttunen R, Hurme M, Aittoniemi J, Huhtala H, Vuento R, Laine J, et al High plasma level of long pentraxin 3 (PTX3) is associated with fatal disease in bacteremic patients: a prospective cohort study. PLoS One. 2011;6: e17653 10.1371/journal.pone.0017653 21423699 PMC3053378

[31] Pettila V, Hynninen M, Takkunen O, Kuusela P, Valtonen M. Predictive value of procalcitonin and interleukin 6 in critically ill patients with suspected sepsis. Intensive Care Med. 2002;28: 1220–1225. 10.1007/s00134-002-1416-1 12209268

[32] Silvestre J, Povoa P, Coelho L, Almeida E, Moreira P, Fernandes A, et al Is C-reactive protein a good prognostic marker in septic patients? Intensive Care Med. 2009;35: 909–913. 10.1007/s00134-009-1402-y 19169668

[33] Lahey T, Shah R, Gittzus J, Schwartzman J, Kirkland K. Infectious diseases consultation lowers mortality from Staphylococcus aureus bacteremia. Medicine (Baltimore). 2009;88: 263–267. 10.1097/MD.0b013e3181b8fccb 19745684 PMC2881213

[34] Nickerson EK, Wuthiekanun V, Wongsuvan G, Limmathurosakul D, Srisamang P, Mahavanakul W, et al Factors predicting and reducing mortality in patients with invasive Staphylococcus aureus disease in a developing country. PLoS One. 2009;4: e6512 10.1371/journal.pone.0006512 19652705 PMC2714962

[35] Honda H, Krauss MJ, Jones JC, Olsen MA, Warren DK. The value of infectious diseases consultation in Staphylococcus aureus bacteremia. Am J Med. 2010;123: 631–637. 10.1016/j.amjmed.2010.01.015 20493464 PMC3606273

[36] Conterno LO, Wey SB, Castelo A. Risk factors for mortality in Staphylococcus aureus bacteremia. Infect Control Hosp Epidemiol. 1998;19: 32–37. 9475347 10.1086/647704

[37] Soriano A, Martinez JA, Mensa J, Marco F, Almela M, Moreno-Martinez A, et al Pathogenic significance of methicillin resistance for patients with Staphylococcus aureus bacteremia. Clin Infect Dis. 2000;30: 368–373. 10671343 10.1086/313650

[38] Johnson LB, Almoujahed MO, Ilg K, Maolood L, Khatib R. Staphylococcus aureus bacteremia: compliance with standard treatment, long-term outcome and predictors of relapse. Scand J Infect Dis. 2003;35: 782–789. 14723349 10.1080/00365540310016682

[39] Jensen AG, Wachmann CH, Espersen F, Scheibel J, Skinhoj P, Frimodt-Moller N. Treatment and outcome of Staphylococcus aureus bacteremia: a prospective study of 278 cases. Arch Intern Med. 2002;162: 25–32. 11784216 10.1001/archinte.162.1.25

[40] Chang FY, Peacock JE Jr, Musher DM, Triplett P, MacDonald BB, Mylotte JM, et al Staphylococcus aureus bacteremia: recurrence and the impact of antibiotic treatment in a prospective multicenter study. Medicine (Baltimore). 2003;82: 333–339. 10.1097/01.md.0000091184.93122.09 14530782

[41] Forsblom E, Ruotsalainen E, Ollgren J, Jarvinen A. Telephone consultation cannot replace bedside infectious disease consultation in the management of Staphylococcus aureus Bacteremia. Clin Infect Dis. 2013;56: 527–535. 10.1093/cid/cis889 23087397

[42] Nagao M, Iinuma Y, Saito T, Matsumura Y, Shirano M, Matsushima A, et al Close cooperation between infectious disease physicians and attending physicians can result in better management and outcome for patients with Staphylococcus aureus bacteraemia. Clin Microbiol Infect. 2010;16: 1783–1788. 10.1111/j.1469-0691.2010.03156.x 21077985

[43] Jensen AG, Espersen F, Skinhoj P, Frimodt-Moller N. Bacteremic Staphylococcus aureus spondylitis. Arch Intern Med. 1998;158: 509–517. 9508229 10.1001/archinte.158.5.509

[44] Osenbach RK, Hitchon PW, Menezes AH. Diagnosis and management of pyogenic vertebral osteomyelitis in adults. Surg Neurol. 1990;33: 266–275. 2326732 10.1016/0090-3019(90)90047-s

[45] Schuetz P, Albrich W, Mueller B. Procalcitonin for diagnosis of infection and guide to antibiotic decisions: past, present and future. BMC Med. 2011;9: 107 10.1186/1741-7015-9-107 21936959 PMC3186747

